# Multi–Year Stability Assessment of Agronomic Performance, Yield and Nutritional Quality of *Bromus inermis* Genotypes in Qinghai Lake Region

**DOI:** 10.3390/plants15101547

**Published:** 2026-05-19

**Authors:** Xin Chen, Wenhui Liu, Wenhu Wang, Wei Hu, Yuhan Wu, Liangrong Zhou, Yilu Liu, Kaiqiang Liu

**Affiliations:** 1 Academy of Animal Science and Veterinary, Qinghai University, Xining 810016, China; chenxinbaafs@163.com (X.C.); wwh01112021@163.com (W.W.); scihw123@163.com (W.H.); wuyuhan9712@163.com (Y.W.); 13299784204@163.com (L.Z.); 15020223182@163.com (Y.L.); 2 Key Laboratory of Qinghai Province Superior Forage Germplasm in the Qinghai–Tibet Plateau, Xining 810016, China

**Keywords:** *Bromus inermis* Leyss., germplasm evaluation, TOPSIS, piecewise structural equation model, genotype × year interaction, forage quality, Qinghai–Tibet Plateau

## Abstract

The reliable identification of productive and nutritionally valuable *Bromus inermis* Leyss. germplasm requires multi–year evaluation because forage performance is strongly influenced by genotype, stand age, and annual environmental variation. We evaluated four experimental genotypes and the cultivar WUSU as a control over three production years at a fixed alpine site on the Qinghai–Tibet Plateau. Agronomic traits, forage yield, dry matter accumulation, and nutritional quality were measured annually. A multi–criteria TOPSIS model was used to integrate yield and quality traits for genotype ranking, while random forest analysis and piecewise structural equation modeling were applied to identify key traits and potential pathways influencing forage performance. Genotype, year, and their interaction significantly affected most agronomic, yield, and nutritional traits. Most traits reached their highest values in the third production year, indicating that this stage was critical for evaluating full productive potential. Among the tested materials, genotype 4–4 showed consistently high biomass production and favorable nutritional performance, whereas WUSU and genotype 1–10 generally ranked lower. Plant height and grass height were positively associated with fresh and hay yield, while fresh forage yield, crude protein content, and stem diameter contributed strongly to model prediction. The SEM results suggested that genotype–year interaction influenced hay yield mainly through changes in stem diameter and acid detergent fiber content. These findings indicate that combining multi–year field evaluation with multi–criteria ranking and pathway analysis can improve the identification of promising *B. inermis* germplasm. Genotype 4–4 represents a useful candidate for further multi–site validation and breeding for high–yield, high–quality forage production in alpine regions. These findings provide a theoretical basis and candidate germplasm for the genetic improvement of *Bromus inermis* Leyss. adapted to the Qinghai–Tibet Plateau.

## 1. Introduction

Perennial grasses are crucial for global livestock production. They not only provide a stable feed supply but also contribute to the sustainability and stability of grassland ecosystems, particularly in unique and fragile environments such as the Tibetan Plateau [1,2,3,4]. *Bromus inermis* Leyss., as a high–quality forage grass, is favored for its high yield, strong stress resistance, and broad ecological adaptability [5]. It is considered one of the most important cool–season grasses in cool and semi–arid regions due to its remarkable cold resistance and its extensive adaptability to various adverse environments, such as cold, drought, and salinity stress [5,6,7]. In contrast to commonly used cool–season grasses like *Poa pratensis* L., which exhibit a significant decline in yield and adaptability under water stress [8], *Bromus inermis* can maintain high forage yield and quality under saline–alkaline and drought conditions, demonstrating greater yield potential and environmental adaptability [9]. Therefore, compared to traditional grasses such as *Elymus sibiricus* L. and *Poa pratensis* L., *Bromus inermis* Leyss. is generally considered to have certain advantages in terms of cold resistance, yield potential, and ecological adaptability [5,9]. These characteristics make it an ideal choice for ecological restoration and pasture construction in high–altitude areas. Although *Bromus inermis* Leyss. exhibits good stress resistance and production potential in cold and semi–arid regions, and has been widely used as excellent forage in various harsh ecological environments [5,10], research and production practices in the Tibetan Plateau region have largely focused on a limited number of local grass species or simple legume–grass intercropping systems [11,12]. Systematic variety breeding and large–scale artificial grassland construction for smooth brome remain relatively limited [13]. The Tibetan Plateau is characterized by long–term low temperatures, high altitude, strong radiation, and frequent seasonal extreme climate events [2]. These harsh environmental conditions significantly increase the risks and costs associated with introducing and cultivating foreign and high–yield varieties over the long term, thereby largely restricting the promotion and application of high–yield and high–quality forage varieties [11,14].

The comprehensive assessment of forage performance is inherently complex because it involves multiple interrelated agronomic and nutritional traits that often differ markedly among genotypes and are strongly influenced by annual environmental fluctuations, manifesting as genotype × year interaction effects [15,16]. Multi–year field trials represent the standard approach for evaluating perennial forage grasses; however, existing studies present three notable limitations that constrain the depth and applicability of genotype evaluation. First, although multi–year trials routinely detect significant genotype × year interactions through an analysis of variance, they rarely integrate the resulting multidimensional trait data into a holistic genotype ranking. Traditional univariate or bivariate statistical methods can identify significant effects for individual traits but do not synthesize these effects across all agronomic and nutritional dimensions to produce a unified assessment, making it difficult to determine which genotype achieves the best overall balance of yield and quality over time [16,17,18]. Second, while previous work has employed correlation–based or descriptive multivariate methods—such as Pearson correlation and simple regression—to identify key yield components in smooth bromegrass [19,20,21], these approaches are fundamentally limited to describing associative relationships and cannot disentangle the trait–mediated associations through which genotype × year interactions are transmitted via intermediate traits (e.g., stem diameter, plant height, fiber content) to ultimately affect forage yield. As a consequence, the mechanistic basis for trait–mediated yield formation in smooth bromegrass remains unclear, limiting the effectiveness of trait–targeted breeding [21,22,23]. Third, existing analytical frameworks generally treat genotype evaluation (ranking) and mechanistic interpretation (pathway analysis) as separate tasks, resulting in a disconnect between identifying which genotype is superior and understanding why it is superior [21,23]. Particularly for the Tibetan Plateau environment, few studies have systematically combined agronomic performance with nutritional quality across consecutive years to address these integrated questions for *Bromus inermis* Leyss. [11,18,24].

The technique for order preference by similarity to ideal solution (TOPSIS) is a multi–criteria decision–making method that compresses multidimensional trait information into a single closeness coefficient, enabling objective genotype ranking based on simultaneous proximity to the ideal solution and distance from the anti–ideal solution [25,26]. However, TOPSIS is inherently a static ranking tool: it identifies which genotype performs the best overall but cannot reveal the trait–mediated mechanisms underlying that performance. Structural equation modeling (SEM), by contrast, quantifies direct and indirect effects among variables through path analysis, thereby capturing the multi–layered putative pathways by which genotype, year, and their interaction influence intermediate traits and, in turn, final yield [27,28]. From a biological standpoint, forage yield formation in smooth bromegrass is a sequential process in which genetic and environmental factors first alter morphological traits such as plant height, stem diameter, and leaf dimensions, which then determine biomass accumulation and nutritional quality [19,21,29]. The path structure of SEM is therefore well suited to representing this hierarchical biological causation. Integrating these two approaches creates a complementary analytical framework: TOPSIS answers the question of “which genotype achieves the best multi–trait performance,” while SEM elucidates “through which trait pathways this genotype achieves superior and stable performance.” This integration advances forage genotype evaluation from associative descriptions to pathway interpretation, yet to our knowledge, no prior study has combined TOPSIS with piecewise SEM for the evaluation of *Bromus inermis* Leyss. genotypes under multi–year field conditions [5,23].

We constructed an evaluation system integrating TOPSIS and piecewise SEM and conducted three consecutive years of fixed–site field observations of four *Bromus inermis* Leyss. accessions and one control variety on the Tibetan Plateau. Specifically, we addressed the following three research questions: (1) Which *Bromus inermis* Leyss. genotype achieves the optimal multi–trait balance of agronomic performance, yield, and nutritional quality across consecutive growing years on the Qinghai–Tibet Plateau? (2) Which phenotypic traits are the primary drivers contributing to hay yield variation among genotypes? (3) Through which putative trait–mediated pathways does the genotype × year interaction influence final dry matter accumulation? By answering these questions, this framework not only enables the precise screening of superior germplasm resources but also provides a mechanistic basis for the genetic improvement of target traits in *Bromus inermis* Leyss. breeding programs.

## 2. Results

### 2.1. Analysis of Agronomic Traits in Different Bromus inermis Genotypes

**Figure 1:**
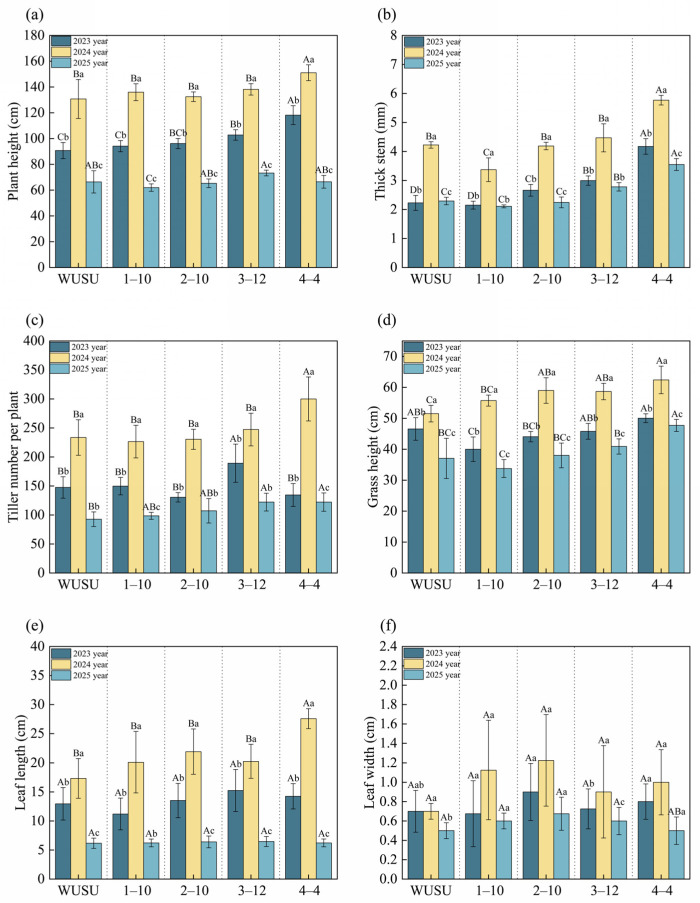
Agronomic traits of different *Bromus inermis* cultivars. (**a**) Plant height (cm); (**b**) Stem thickness (mm); (**c**) Tiller number per plant; (**d**) Grass height (cm); (**e**) Leaf length (cm); (**f**) Leaf width (cm). Note: Performance of major agronomic traits of *Bromus inermis* genotypes across different planting years. Bars show mean values, and error bars indicate mean ± SE. Different uppercase letters indicate significant differences among genotypes within same year, whereas different lowercase letters indicate significant differences between years within same genotype at *p* < 0.05. Year 2 = 2023 (second production year after sowing in June 2022); year 3 = 2024 (third production year); year 4 = 2025 (fourth production year).

We evaluated the agronomic traits and annual consistency of four *Bromus inermis* Leyss. genotypes (1–10, 2–10, 3–12, 4–4) compared with the control genotype WUSU. This experiment was conducted over three production years at a fixed field site. The main agronomic indexes such as the plant height, stem diameter, tiller number per plant, grass height, leaf length and leaf width of *Bromus inermis* Leyss. were determined continuously in the second to fourth years. A two–way ANOVA showed that stem diameter, plant height, tiller number per plant, canopy height, leaf length, and leaf width were all significantly affected by genotype, year, and genotype × year interaction (*p* < 0.05) (Table S1). In addition, the interaction between genotype and planting years had significant effects on most traits, emphasizing the decisive significance of these factors on the characteristics of *Bromus inermis*. There were significant differences between genotypes and years, and most traits peaked in the third year and declined in the fourth year (Figure 1). In the third year, genotype 4–4 showed significant advantages in plant height (151 cm), stem diameter (5.8 mm), number of tillers per plant (300), leaf length (28 cm), and leaf width (1.2 cm) (Figure 1a–c,e,f). The control genotype WUSU showed poor performance in most traits. These results highlight the key interaction between genotype and year, indicating that genotype 4–4 is a consistently high–performing and preferred choice.

### 2.2. Yield Analysis of Different Genotypes of Bromus inermis

**Figure 2:**
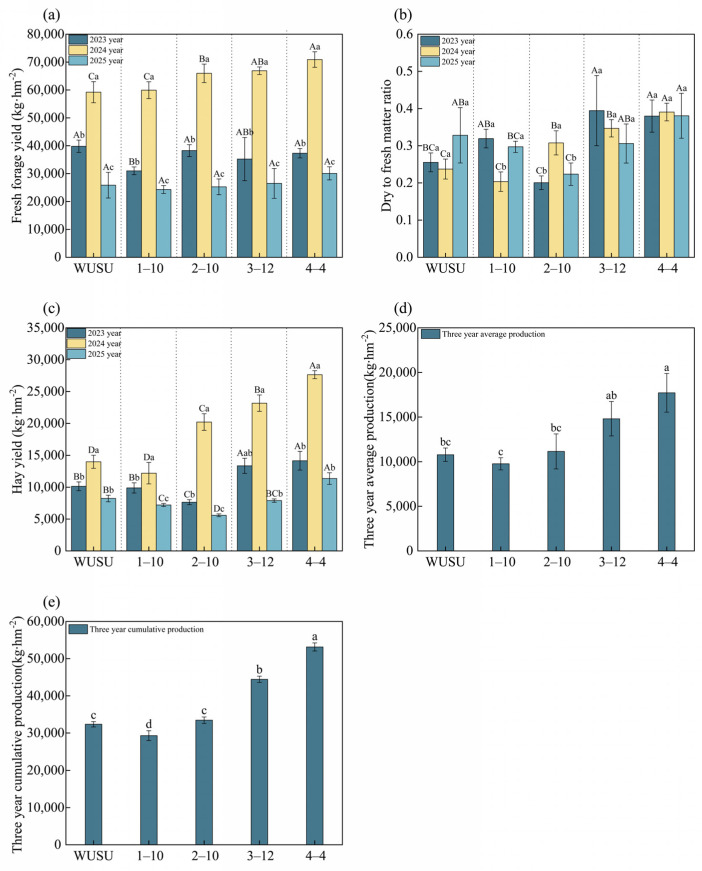
The yield of different varieties of *Bromus inermis*. (**a**) Fresh forage yield (kg·hm^−2^) across three years (2023, 2024, and 2025); (**b**) Dry-to-fresh matter ratio across three years (2023, 2024, and 2025); (**c**) Hay yield (kg·hm^−2^) across three years (2023, 2024, and 2025); (**d**) Three-year average production (kg·hm^−2^); (**e**) Three-year cumulative production (kg·hm^−2^). Note: Bars show mean values, and error bars indicate mean ± SE. Different uppercase letters indicate significant differences among genotypes within same year, whereas different lowercase letters in-dicate significant differences between years within same genotype at *p* < 0.05. Year 2 = 2023 (second production year after sowing in June 2022); year 3 = 2024 (third production year); year 4 = 2025 (fourth production year).

We compared fresh forage yield, dry matter content and hay yield among five genotypes of *Bromus inermis* Leyss. for three years. An ANOVA (*p* < 0.05) showed that genotype and year had major main effects on yield. Additionally, hay yield was also strongly related to genotype by year. All varieties were highly productive for three years, and productivity reached a peak in the third growing year. Genotype 4–4 performed well in all yield parameters, achieving the highest fresh yield of 70,917 kg∙hm^−2^ (Figure 2a); highest hay yield of 3 years of 27,681 kg∙hm^−2^ (Figure 2c); highest three–year average of 17,716 kg∙hm^−2^ (Figure 2d); and highest cumulative hay yield of 53,148.7 kg∙hm^−2^ (Figure 2e). The dry-to-fresh matter ratio of genotype 4–4 was consistently high and remained consistently high at a constant level between 0.38 and 0.39 over the three years (Figure 2b). Genotype 3–12 ranked second in cumulative hay yield, with 44,443 kg·hm^−2^, which remained lower than that of genotype 4–4 (Figure 2e). Genotype 2–10 had very large changes between years in terms of dry matter content and hay yield, while control WUSU and genotype 1–10 produced low–level yields with moderate stability. Collectively, our results show that genotype 4–4 is a high–yielding and consistently high–performing genotype, with its performance strongly depending on the growing year. It is a good candidate for forage systems demanding productivity and reliability.

### 2.3. Nutritional Analysis of Different Genotypes of Bromus inermis

**Figure 3:**
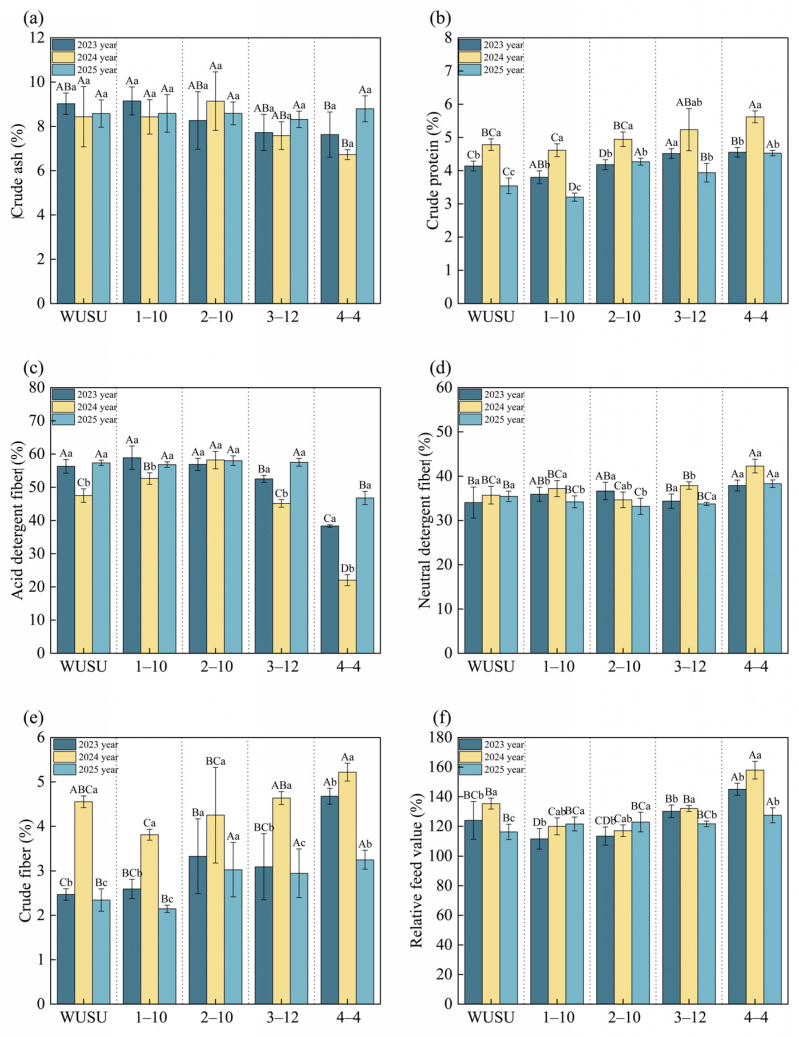
Nutritional qualities of different *Bromus inermis* genotypes. (**a**) Crude ash (%); (**b**) Crude protein (%); (**c**) Acid detergent fiber (%); (**d**) Neutral detergent fiber (%); (**e**) Crude fiber (%); (**f**) Relative feed value. Note: Bars show mean values, and error bars indicate mean ± SE. Different uppercase letters indicate significant differences among genotypes within same year, whereas different lowercase letters in-dicate significant differences between years within same genotype at *p* < 0.05. Year 2 = 2023 (second production year after sowing in June 2022); year 3 = 2024 (third production year); year 4 = 2025 (fourth production year).

Genotype, planting year, and their interaction significantly affected the nutritional quality indicators of *Bromus inermis* Leyss. (*p* < 0.05) (Table S2). This highlighted the importance of genotype selection and planting year in determining forage nutritional value. The crude protein content of each genotype exhibited notable inter–annual variation, peaking in the third year before declining in the fourth year. These findings underscored the significant impact of harvest year and genotype on all nutritional quality traits. Consistently, several key nutritional indicators, particularly CP and RFV, improved in the third production year, although not all quality–related traits followed the same direction, followed by a decline in the fourth year (Figure 3). In particular, the crude fiber content and RFV of genotype 4–4 peaked in the third year at 5.6% and 158.0, respectively, compared to 4.6% and 145.0 in the second year, and decreased to 4.5% and 127.5% in the last year (Figure 3e,f). Among the genotypes, 4–4 outperformed others in multiple traits during the third year, with a higher crude protein content (5.6%, compared to 4.6% to 5.2% in others) and a lower ADF content (22.0%, compared to 45.0% to 58.0% in others, Figure 3b,c). Notably, genotype 2–10 maintained consistently high ADF levels (57.0–58.0%) across years, indicating stable but relatively unfavorable fiber accumulation from the perspective of digestibility. However, the relatively high NDF content of genotype 4–4 (42.0% in the third year, compared to 34.0% to 37.0% in others) might partially offset its nutritional benefits (Figure 3d). While the crude ash content remained consistently high over time in most genotypes, it significantly increased in genotype 4–4 in the fourth year (Figure 3a). Overall, the results emphasized that the third growing year was optimal for nutritional quality, likely due to physiological maturity, while the subsequent decline might be attributed to age–related senescence or environmental stress.

### 2.4. Comprehensive Evaluation of Different Bromus inermis Resources

To identify the *Bromus inermis* Leyss. genotype with the best overall multi–trait performance, we applied a three–step analytical framework combining the Mantel test, K–means clustering, and TOPSIS evaluation.

**Figure 4:**
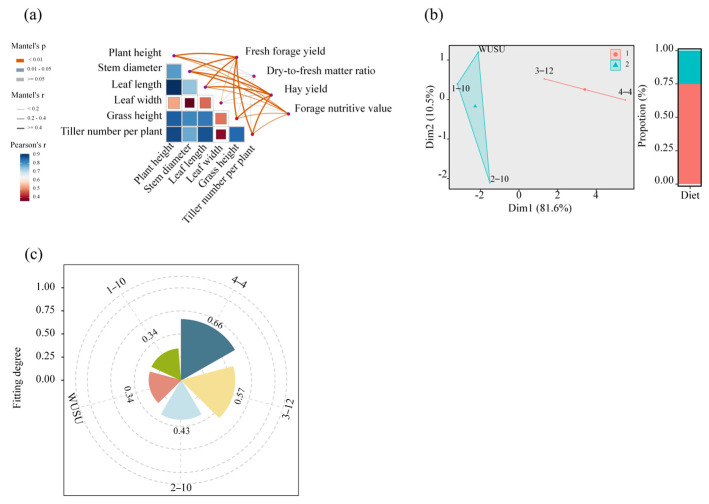
Multi–method evaluation of five *Bromus inermis* genotypes across three production years. (**a**) Mantel test heatmap showing associations between morphological traits and yield/quality dimensions; curve color denotes Mantel correlation coefficient (r), and curve width denotes statistical significance. (**b**) K–means clustering visualized on first two principal components (Dim1 = 81.6%; Dim2 = 10.5%); Group 1 = genotypes 3–12 and 4–4 (high–performance); Group 2 = genotypes WUSU, 1–10, and 2–10 (lower–performance). (**c**) TOPSIS ranking of five genotypes by closeness coefficient (C_i_); higher C_i_ indicates better overall multi–trait performance.

The Mantel test (Figure 4a) was performed to assess the associations between key agronomic traits and production–related indicators of *Bromus inermis* Leyss. The correlation matrix revealed strong positive associations among plant height, leaf length, tiller number per plant, grass height, and stem diameter (*r* = 0.70–0.90), whereas leaf width showed comparatively weaker correlations. Both fresh forage yield and hay yield were strongly influenced by plant height, leaf length, and tiller number (*r* ≥ 0.40, *p* < 0.01), and significantly affected by stem diameter and grass height (*p* < 0.05), but were not associated with leaf width. Nutritional quality showed highly significant correlations with plant height and leaf length (*p* < 0.01) and significant correlations with tiller number, stem diameter, and grass height (*p* < 0.05), whereas the dry–to–fresh ratio was only moderately correlated with plant height and leaf length (0.01 ≤ *p* < 0.05). Collectively, plant height and leaf length emerged as the core agronomic traits coordinately regulating both yield and quality. The K–means clustering of the standardized three–year multi–trait dataset partitioned the five genotypes into two well–separated groups along the first two principal components (Dim1 = 81.6%; Dim2 = 10.5%; Figure 4b): Group 1 comprised the high–performance genotypes 3–12 and 4–4, while Group 2 included the lower–performance genotypes WUSU, 1–10, and 2–10. Independent–sample *t*–tests (Table 1) provided supporting evidence for phenotypic divergence between the two clusters, revealing significant between–group differences in stem diameter (*t* = 4.6, *p* < 0.001), grass height (*t* = 2.6, *p* = 0.01), hay yield (*t* = 3.5, *p* = 0.001), crude protein (CP, *t* = 3.6, *p* = 0.001), acid detergent fiber (ADF, *t* = −4.9, *p* < 0.001), and relative feed value (RFV, *t* = 5.1, *p* < 0.001), confirming that the clustering captured biologically meaningful divergence between the two performance groups.

The entropy–weighted TOPSIS method subsequently compressed all 15 indicators into a single closeness coefficient (C_i_; Figure 4c, Table 2 and Table 3). Genotype 4–4 ranked first (C_i_ = 0.66), positioning it the closest to the ideal solution, followed by 3–12 and 2–10 with intermediate–to–high scores, whereas WUSU and 1–10 ranked the lowest. The TOPSIS ranking fully converged with the K–means partition, as both Group 1 genotypes occupied the top two positions, reinforcing the robustness of the evaluation. Collectively, these convergent analyses identify plant height and leaf length as the core yield– and quality–regulating traits and genotype 4–4 as the top performer, providing both a robust ranking for germplasm selection and clear trait–based criteria for breeding *Bromus inermis* Leyss. adapted to the Qinghai–Tibet Plateau.

### 2.5. Essential Elements Influencing the Production Efficacy of Bromus inermis Resources

**Figure 5:**
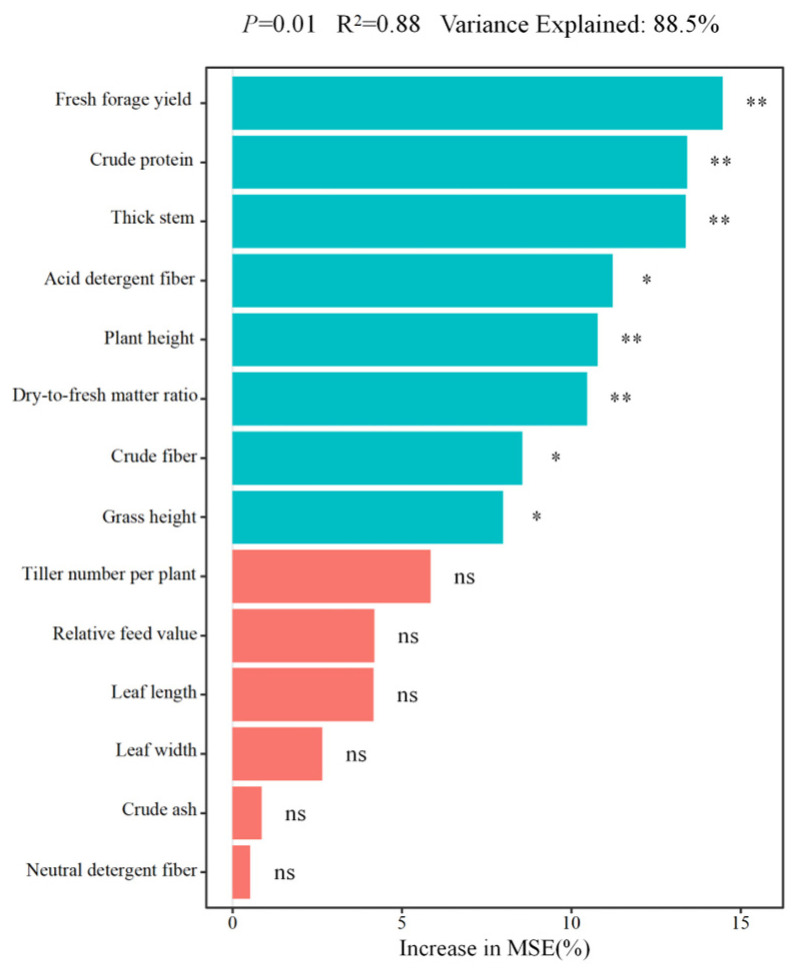
Random forest variable importance ranking for predictors of hay yield in *Bromus inermis*. Note: Asterisks indicate the statistical significance of the permutation test for variable importance * *p* < 0.05, ** *p* < 0.01, while the bar length represents the magnitude of each variable’s contribution to model accuracy (%Increase in MSE). These two dimensions should be interpreted independently: a variable with a single asterisk may still contribute substantially to model prediction if its %Increase in MSE is high.

In order to determine the primary factors impacting model accuracy, we conducted a contribution analysis to assess the influence of each trait on the mean squared error (MSE) (Figure 5). The piecewise SEM was parameterized based on the top–ranking variables identified by the random forest importance analysis so that the causal pathways tested were empirically grounded rather than arbitrarily specified. The analysis showed that fresh forage yield, crude protein content, and stem diameter were the three largest contributors, with contribution percentages of 14.5%, 13.4%, and 13.3%, respectively. Other traits—acid detergent fiber, plant height, dry–to–fresh matter ratio, fiber content, and grass height—also had substantial effects, with contribution percentages ranging from 8.0% to 11.2%. Although ADF reached only a moderate significance level (*p* = 0.02, marked with a single asterisk), its contribution percentages (11.2%) ranked fourth among all 14 predictors, indicating that ADF is a quantitatively important but moderately variable predictor of hay yield. The model demonstrated high predictive performance, yielding an R^2^ of 0.88, a variance explained of 88.54%, and a statistically significant *p*–value of 0.01. These results indicated that the listed traits played a substantial role in hay yield formation.

### 2.6. The Impact Process and Pathways of Bromus inermis Forage Yield

**Figure 6:**
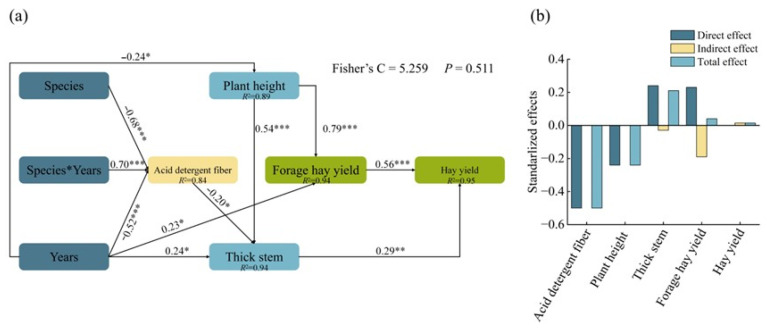
A piecewise structural equation model showing the direct and indirect relationships among genotype, planting year, agronomic traits, and forage yield in *Bromus inermis*. (**a**) Standardized path coefficients for the final SEM. (**b**) The direct, indirect, and total effects of the major explanatory variables on hay yield. * *p* < 0.05, ** *p* < 0.01, *** *p* < 0.001.

To elucidate the pathways through which genotype, planting year, and key agronomic traits influenced forage production in *Bromus inermis* Leyss., a piecewise structural equation model (SEM) was constructed (Figure 6a). The SEM revealed that the genotype × year interaction is an important upstream factor affecting yield through two major pathways. On the one hand, genotype × year negatively affected stem diameter (−0.52), whereas stem diameter had a positive direct effect on hay yield (0.29). On the other hand, genotype × year positively affected ADF (0.70), and ADF negatively affected hay yield (−0.29). In addition, genotype negatively affected plant height (−0.24), while plant height exerted strong positive effects on forage hay yield (0.54) and total hay yield (0.79). The model showed a satisfactory overall fit (Fisher’s C = 5.3, *p* = 0.51), supporting the adequacy of the hypothesized pathway structure. A further decomposition of effects showed that stem diameter had a positive total effect (0.21) on hay yield (Figure 6b). Taken together, these results indicate that genotype– and year–related variation affected forage yield mainly through coordinated changes in structural traits and forage quality, with stem diameter and plant height promoting yield formation and ADF exerting a negative influence. From a breeding perspective, these findings suggest that selection for larger plant size, thicker stems, and lower ADF may contribute to improved forage productivity in *Bromus inermis*, while the interaction between genotype and annual environmental conditions should also be considered.

## 3. Discussion

**Figure 7:**
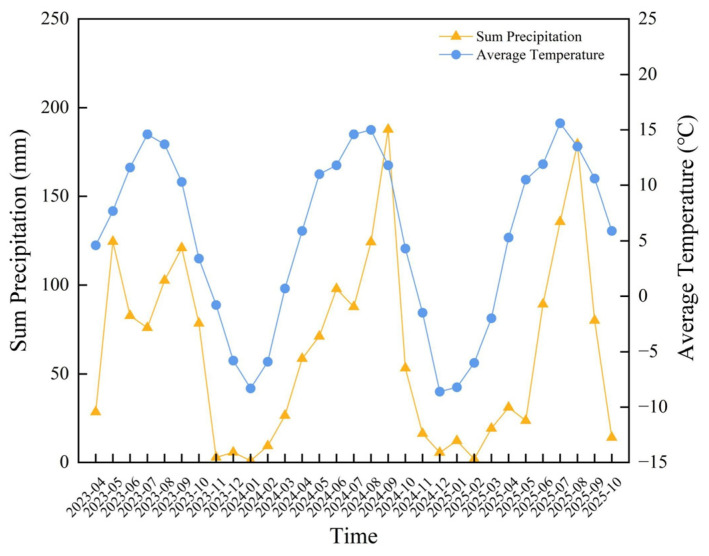
The monthly average temperature and precipitation distribution at the experimental site for the period spanning from April 2023 to October 2025.

To systematically evaluate and interpret genotype performance, we employed a two–stage analytical framework in which TOPSIS provided a holistic genotype ranking and piecewise SEM subsequently elucidated the mechanistic pathways underlying that ranking, together constituting an integrated approach of evaluation followed by causal interpretation. The temporal pattern observed in our trial, peak productivity in the third production year followed by a decline in the fourth, aligns with the developmental trajectory reported across multiple long–term studies of *Bromus inermis* Leyss. *Bromus inermis* Leyss. generally establishes slowly in the first year, with maximum yields typically attained in the third or fourth year of cultivation [5,19,30]. Mackiewicz–Walec et al. [5], reviewing over 50 years of smooth brome cultivation records, reported that biomass typically peaks between years 3 and 5, with the subsequent decline driven by sod–bound rhizome aging. Lardner et al. [20] documented a 15–22% decline in hay yield between years 4 and 6 in Saskatchewan trials, while Ou et al. [29] further demonstrated that without rejuvenation practices, *Bromus inermis* stands exhibit progressive vigor loss after year 4. These convergent findings support our interpretation that the year 4 decline reflects an intrinsic developmental trajectory rather than an artifact of our specific trial conditions. The third–year peak observed in our trial likely reflects a convergence of two factors: physiological maturity, characterized by a fully established root system and maximum tiller density, and favorable growing–season precipitation recorded in 2024 (Figure 7). The fourth–year decline is consistent with the well–documented sod–bound phenomenon in *Bromus inermis*, whereby intertiller competition and rhizome aging reduce stand vigor [5]. Disentangling these two drivers would require controlled irrigation experiments, which we identify as a priority for future research. Under favorable conditions with multiple harvests per season, *Bromus inermis* Leyss. can produce up to approximately 13 t∙ha^−2^ of dry matter annually at full stand maturity [20]. The substantially higher hay yields observed for genotype 4–4 in its third year reinforce *Bromus inermis’s* capacity for prolific biomass production under appropriate agronomic management [5,9,31] and directly address our research question regarding annual productivity stability by identifying the third production year as the critical window for evaluating full yield potential. Long–term trials (≥6 years) of these specific Qinghai–Tibet Plateau–adapted genotypes have not yet been published, underscoring the value of our three–year dataset as a foundation for future extended evaluations. Extending the trial to six or more production years would clarify whether genotype 4–4 maintains its superiority beyond the establishment–peak–decline cycle and would enable the separation of climatic anomaly effects from intrinsic stand–aging dynamics.

Beyond yield, we also obtained insights into nutritional value and feeding utility that both align with and extend prior findings. Genotype 4–4 combined high crude protein with moderately elevated neutral detergent fiber (NDF), illustrating a typical trade–off between forage quality and biomass accumulation that has been widely reported in perennial forages [5]. Lardner et al. [20] found that as *Bromus inermis* Leyss. and related bromes advanced in growth stage, crude protein and digestibility declined, while fiber concentrations (NDF and ADF) increased. Despite its relatively higher NDF, genotype 4–4 still achieved the highest relative feed value (RFV ≈ 158 in year 3), indicating that its superior crude protein content offset the fiber disadvantage. The combination of low ADF and moderately elevated NDF in genotype 4–4 suggests a fiber profile rich in hemicellulose but low in lignin, which is favorable for ruminant digestion. Because NDF determines voluntary intake potential while ADF governs digestibility, the low ADF in genotype 4–4 ensures high estimated dry matter digestibility (DDM = 71.8%), and the resulting RFV of 158 classifies this forage as “Prime” quality according to established hay grading standards [32], confirming its practical suitability as high–quality roughage in ruminant diets. These results are consistent with the generally high feeding value reported for *Bromus inermis* Leyss., a species recognized for excellent palatability and digestibility owing to its inherently moderate fiber levels and relatively high protein of good biological value [5,33]. Previous grazing experiments conducted in Western Canada demonstrated that *Bromus inermis* pastures delivered nutritional benefits and enhanced beef cattle performance comparable to other improved bromegrass cultivars [19,34]. The present results extend this evidence by demonstrating that targeted genotype selection (genotype 4–4) can further enhance both yield and feed quality simultaneously. The relatively modest CP content observed (peaking at 5.6%) should be interpreted within the context of the extreme alpine environment of the trial site, where nitrogen mineralization is inherently slow and no supplemental nitrogen was applied. Under comparable high–altitude conditions, CP values of 4–8% in cool–season grasses are considered typical [5,24], indicating that genotype 4–4 approached the upper range achievable without nitrogen fertilization.

A significant finding of the present work was the robust relationship between the morphological characteristics of plants and their productivity in forage, investigated through both empirical correlations and structural modeling techniques. Plant height was positively related to fresh and hay yields, and stem diameter emerged as a major determinant of final hay yield. These results were consistent with previous reports and provided a mechanistic perspective on how genotype–environment interactions shaped yield [16,35,36]. The path analyses in *Bromus inermis* Leyss. showed that plants with taller stature and greater stem abundance (or higher stem/culm density) tended to produce higher seed yields, identifying stem density and plant height as primary selection criteria for yield improvement [21,22,29].

Using the piecewise SEM, we obtained a more mechanistic interpretation of forage yield formation in *Bromus inermis* Leyss. by separating the direct and indirect pathways linking genotype, planting year, and key agronomic traits to yield. In the present study, structural traits, particularly plant height and stem diameter, were positively associated with forage production, indicating that yield improvement in *Bromus inermis* depends largely on traits related to plant size and biomass support. This interpretation is biologically reasonable, as taller plants generally have greater aboveground growth potential, while thicker stems contribute to mechanical support and dry matter accumulation, thereby favoring higher hay yield. By contrast, the SEM suggested that the effects of genotype × year were expressed mainly through indirect pathways rather than through a single direct effect on yield. Specifically, the negative pathway from genotype × year to stem diameter, together with the positive effect of stem diameter on hay yield, indicates that annual environmental variation may modify the yield performance of different genotypes by altering stem development. Likewise, the positive effect of genotype × year on ADF, combined with the negative effect of ADF on hay yield, suggests that some genotype–environment combinations may reduce productivity through increased structural fiber concentration. This distinction between direct and indirect effects is important because it shows that genotype and planting year do not influence forage yield independently of plant traits but rather through coordinated changes in morphology and forage quality [23,37,38]. The statistical modeling results are biologically meaningful because they help distinguish traits that are merely correlated with yield from those that may play a more central role in yield formation. For example, plant height likely reflects canopy expansion and light interception capacity, whereas stem diameter may contribute to structural support and biomass accumulation. In contrast, higher ADF generally indicates greater fiber deposition and reduced digestible biomass, which may explain its negative association with hay yield in the present study. Therefore, the combined use of random forest and piecewise SEM provides not only a statistical ranking of trait importance but also a biologically interpretable framework for understanding how multiple traits jointly influence forage productivity. Despite the usefulness of the integrated modeling framework, several limitations should be considered when interpreting the present results. It should be noted that the genetic panel included only five genotypes (four experimental accessions plus one control), which constrained the statistical power for detecting genotype effects and limited the representativeness of the results to the broader *Bromus inermis* germplasm pool. The findings, particularly the TOPSIS rankings, should therefore be interpreted as preliminary screening outcomes rather than definitive genotype recommendations, and validation with a larger and more diverse panel is warranted. First, the present work was based on a limited number of genotypes under a specific set of environmental conditions and over a finite number of planting years; therefore, caution is needed when generalizing these findings to other germplasm resources, ecological regions, or management systems. Second, although random forest and piecewise structural equation modeling were effective for identifying key traits and their potential pathways of influence, these approaches are primarily inferential and do not establish causality definitively. The observed trait–yield relationships should therefore be interpreted as biologically plausible associations that require further validation across broader populations and multi–environment trials. Third, some unmeasured factors, such as belowground traits, disease pressure, and micro–environmental heterogeneity, may also contribute to variation in forage productivity but were not explicitly included in the present analysis. Future studies incorporating broader germplasm, additional environments, and independent validation datasets will be important for testing the robustness and general applicability of the conclusions.

Although some of the observed patterns are broadly consistent with previous reports on forage development and maturity, the present work provides important new insights into yield formation in *Bromus inermis* Leyss. Rather than considering agronomic and quality traits separately, our results show that forage productivity is shaped by coordinated interactions among structural traits, fiber–related traits, and genotype × year effects. By integrating random forest analysis with piecewise structural equation modeling, we identified not only the most influential traits associated with forage yield but also their direct and indirect pathways. In particular, plant height and stem diameter contributed positively to forage production, whereas ADF was negatively associated with hay yield. These findings indicate that selection for high–performing genotypes should consider both trait values and their stability across years. Because both yield and quality peaked in the third year, the future breeding of *Bromus inermis* Leyss. should prioritize sustained productivity, including traits such as improved tiller renewal capacity and stress or disease resistance that may support long–term pasture performance. In addition, phenotypes with greater plant height, thicker stems, and lower fiber accumulation may serve as practical selection targets for simultaneously improving yield and forage quality. The multi–criteria evaluation approach used here, including TOPSIS, also proved useful for identifying genotypes with balanced performance across multiple traits. More broadly, the multi–year evaluation and structural modeling framework developed in the present work provides a useful template for dissecting genotype–environment interactions in other perennial forage species and may support more efficient and sustainable forage breeding.

## 4. Materials and Methods

### 4.1. Experimental Site Description

This research was conducted at the National Perennial Pasture Germplasm Nursery, located in Xihai Town, Qinghai Province (100°52.848′ E, 36°59.36′ N). With an altitude of 3156 m, this location exemplifies a high–altitude alpine environment. The region showed a distinct alpine climate, characterized by an average yearly sunshine duration of 2980 h and a mean annual temperature of only 0.9 °C, which reflects notably low–temperature conditions. In January, temperatures could plummet to –27.3 °C, while in August, they reached only 25 °C. The region had a brief frost–free period averaging 93 days annually, with frost events possible year–round. Precipitation varied seasonally, averaging 369.1 mm annually, mostly falling between July and September (Figure 7). The annual evaporation rate was high at 1400 mm, characteristic of a cold–arid climate. Regarding soil properties, the pH in the 0–20 cm soil layer was 8.43, indicating alkaline conditions. The soil contained 32.48 g·kg^−1^ of organic matter, 1.56 g·kg^−1^ of total nitrogen, and 1.39 g·kg^−1^ of total phosphorus. Available phosphorus measured 2.2 mg·kg^−1^, and available nitrogen was 88.8 mg·kg^−1^ [39].

### 4.2. Experimental Design

The plant materials used in this experiment were four wild *B. inermis* accessions (1–10, 2–10, 3–12, and 4–4) previously collected from the Qinghai–Tibet Plateau, together with the cultivar WUSU as a control. Detailed information on the morphological characteristics, productivity range, and ecological adaptation of each accession is summarized in Table 4. Subsequent to the classification and screening conducted in the field, four wild accessions of *Bromus inermis* Leyss. (numbers 1–10, 2–10, 3–12, and 4–4), characterized by high productivity and strong adaptability, were selected for the trial, with WUSU as the control. This experiment was initiated in early June 2022. Before the sowing process, the experimental site underwent thorough plowing and leveling, with materials like stones and root systems eliminated. The trial took place over three consecutive years, utilizing a completely randomized block design comprising four replications. To maximize the production capabilities of the tested materials, a single–plant cultivation approach was employed. Initially, seeds were placed in seedling trays (specifications: outer dimensions of 540 mm × 280 mm; containing 105 cells; with an upper aperture of 35 mm, lower aperture of 10 mm; and a depth of 40 mm; each cell having a volume of 25 mL) to facilitate germination. A mixture of garden soil and nutrient substrate in a 1:1 ratio was used to fill the trays, and germination occurred within a greenhouse environment. To improve the survival rate of the seedlings, the trays were relocated to the experimental field for the hardening process following germination. Once the seedlings developed to the two–leaf–one–heart stage, they were transplanted into the field. Both the planting spacing and row spacing within the experimental area measured 80 cm, and each experimental plot covered an area of 3 m × 5 m. Surrounding the experimental field, a protective belt measuring 1.5 m wide was planted with WUSU, along with a 1 m wide walkway established between the plots. Single–plant planting is a common strategy in the primary screening stage of germplasm resources. Its purpose is to eliminate inter–individual competition effects, allowing for the full expression of each genotype’s genetic potential, thereby enabling a more accurate assessment of intrinsic differences between genotypes [40]. Diammonium phosphate ((NH_4_)_2_HPO_4_) was utilized as a base fertilizer at a dosage of 75 kg per hectare. Following the establishment of the experimental seedlings, activities such as irrigation, fertilization, and grazing were restricted. Standard field management practices were carried out: weeding occurred three times during the initial year (2022), whereas in the subsequent years (year 2 = 2023 (second production year after sowing in June 2022); year 3 = 2024 (third production year); year 4 = 2025 (fourth production year)), two intertillage weeding sessions were carried out after green–up and prior to stem elongation. Additionally, measures for controlling plant diseases, pests, and rodents were implemented [40].

### 4.3. Indicator Determination

#### 4.3.1. Agronomic Trait Assessment

Sampling and indicator measurements were conducted during the flowering phase in August 2023 (second production year after sowing in June 2022), 2024 (third production year), and 2025 (fourth production year). Within each experimental plot, ten plants demonstrating comparable growth vigor and showing no significant signs of disease or pest infestation were chosen at random. Measurements for plant height (from the ground to the highest point) and grass height (from the ground to the tip of the highest leaf) for each accession were taken using a measuring tape. The number of tillers, which are shoots emerging from the base of the main stem, was counted for every plant. Additionally, both the length and width of the flag leaf, which is the largest section of the leaf blade, were assessed. Forage yield evaluation took place during the early flowering stage of *Bromus inermis* Leyss. A 2 m × 2 m quadrat was selected in each plot, and the vegetation was cut at a stubble height of 5 cm. After measuring the fresh weight, a 1000 g sample from each plot was placed in a mesh bag and brought indoors. The samples were deactivated at 105 °C and then dried at 75 °C until a constant weight was achieved. The dry weight was measured, and the hay yield was calculated accordingly [40,41].

#### 4.3.2. Nutritional Indicator Measurement

For each sampling site, we gathered fresh and clean forage leaves, noting their initial weights. Following air–drying, the forage samples were placed in a forced–air oven set to 60 °C for an additional 24 h. Once dried, we ground the samples and sieved them through a 2 mm screen. The plant dry matter (DM) was assessed by drying the samples at 105 °C until they reached a consistent weight. To measure total ash content, we applied high–temperature ashing in accordance with AOAC protocols, heating the samples in a muffle furnace at 550 °C until a stable weight was obtained. Nitrogen (N) was determined utilizing a modified semimicro Kjeldahl method [42]. Crude protein (CP) was calculated by multiplying the N content obtained with a factor of 6.25. Neutral detergent fiber (NDF) and acid detergent fiber (ADF) were measured following the protocol established by Van Soest et al., while crude fat was derived from the ether extract (EE) analysis [43]. Lastly, relative feed value (RFV) was estimated based on the calculated dry matter digestibility (DDM) and dry matter intake (DMI) [31]:

DDM % = 88.9 − (0.779 × %ADF),

DMI % of BW = 120/%NDF,

RFV = (%DDM × %DMI)/1.29,

ADF: Acid detergent fiber (% of DM).

DMI: Dry matter intake (% of body weight).

### 4.4. Statistical Analysis

#### 4.4.1. Data Preprocessing and Assumption Testing

The initial data organization, unit conversion, and per–plant trait calculation were performed in Microsoft Excel 2019 (Microsoft Corporation, Redmond, WA, USA). Prior to parametric analysis, all trait variables were screened for distributional assumptions using SPSS 19.0 (IBM Corporation, Armonk, NY, USA). Specifically, the normality of each trait within every genotype × year combination was assessed by the Kolmogorov–Smirnov test with Lilliefors significance correction [44], and homogeneity of variance across groups was evaluated by Levene’s test based on deviations from the group median rather than the mean, as this formulation is more robust to non–normality [45]. Both tests were judged at the α = 0.05 significance level. For trait distributions that marginally violated normality (0.01 < *p* < 0.05 in the Kolmogorov–Smirnov test), we additionally inspected skewness and kurtosis values; all such cases fell within the acceptable range of |skewness| < 2 and |kurtosis| < 7, supporting the robustness of subsequent ANOVA procedures [46]. No data transformations were applied because all traits satisfied the combined assumption criteria.

#### 4.4.2. Analysis of Variance and Multiple Comparisons

Following the validation of the distributional assumptions, a one–way analysis of variance (ANOVA) was conducted separately for each year to test for genotype effects on each measured trait, using genotype (five levels: four accessions plus one control variety) as a fixed factor. When the omnibus F–test indicated significant among–genotype variation (*p* < 0.05), post hoc pairwise comparisons were performed using Fisher’s least significant difference (LSD) method at the 0.05 significance threshold [47,48]. The LSD method was selected because the number of treatment levels was small (k = 5) and the experimental design was balanced, conditions under which the LSD provides adequate Type I error control while maintaining statistical power [46]. In addition, a two–way factorial ANOVA was performed with genotype and year (three levels: 2023, 2024, 2025) as fixed factors and their interaction (genotype × year) as an additional term to quantify the relative contributions of genetic, environmental, and interactive sources of variation to each agronomic, yield, and nutritional quality trait. Effect sizes were reported as partial eta–squared (partial η^2^) to facilitate a comparison of the magnitude of each factor’s contribution across traits [48].

#### 4.4.3. K–Means Cluster Analysis

K–means cluster analysis was applied to classify the five *Bromus inermis* Leyss. accessions based on their multi–trait performance profiles. Prior to clustering, all trait variables were standardized to zero mean and unit variance (z–score transformation) to eliminate the influence of differing measurement scales. The optimal number of clusters (k) was determined by the elbow method, which identifies the inflection point in the plot of the total within–cluster sum of squares against k, supplemented by the average silhouette width criterion; the value of k that maximized the mean silhouette coefficient was selected [46]. The K–means algorithm was initialized using the k–means++ method to improve convergence stability and was run with 25 random restarts (nstart = 25) to minimize the risk of converging to a local optimum. The maximum number of iterations per run was set to 300 (iter.max = 300). Cluster analysis and visualization were performed using the factoextra (version 1.0.7) and ggplot2 (version 3.4.0) packages in R 4.0.2 (R Core Team, Vienna, Austria).

#### 4.4.4. Random Forest Variable Importance Analysis

A random forest regression model was constructed to evaluate the relative importance of all measured predictor variables in explaining variation in hay yield per plant. The predictor set comprised six agronomic traits (plant height, stem diameter, tillers per plant, grass layer height, leaf length, leaf width), six nutritional quality indicators (crude protein, acid detergent fiber, neutral detergent fiber, crude fat, crude ash, relative feed value), and two yield–related parameters (fresh grass yield per plant, dry–to–fresh ratio), totaling 14 predictors. A full–variable inclusion strategy was adopted without prior screening because the random forest algorithm inherently accommodates multicollinearity through its ensemble–based decorrelation mechanism and provides built–in variable importance rankings, thereby eliminating the need for preliminary collinearity diagnostics or stepwise selection [47].

The model was parameterized as follows: the number of trees (ntree) was set to 1000 to ensure stable importance estimates, and the number of candidate variables randomly sampled at each split (mtry) was set to the default value for regression, which equals the floor of the total number of predictors divided by three (mtry = ⌊14/3⌋ = 4). The minimum node size for terminal nodes was set to 5 (nodesize = 5), the default for regression forests. Model significance was assessed using a permutation–based test implemented in the rfUtilities package (version 2.1–5): the observed model pseudo–R^2^ was compared against a null distribution generated from 1000 permutations of the response variable, and model–level significance was judged at α = 0.05. Variable–level significance was similarly evaluated using the rfPermute package (version 2.5.1), which generated null distributions of variable importance scores (percentage increase in mean squared error, %IncMSE) through 1000 permutations per variable; variables with permutation *p* < 0.05 were deemed significantly important. All random forest analyses were conducted using the randomForest package (version 4.7–1.1) in R 4.0.2, with the random seed set to 123 (set. seed (123)) to ensure reproducibility.

#### 4.4.5. Piecewise Structural Equation Modeling

A piecewise structural equation model (piecewise SEM) was constructed to quantify the direct and indirect pathways through which genotype, planting year, and their interaction influenced hay yield per plant in *Bromus inermis* Leyss. The piecewise SEM approach decomposes a complex multivariate causal structure into a set of individual linear models, each corresponding to a single endogenous variable in the path diagram, and evaluates overall model fit from the combined set of component models [26,27]. This approach was preferred over traditional global SEM (e.g., maximum–likelihood–based covariance structure analysis) for three reasons: it accommodates small sample sizes more robustly, permits the inclusion of different distributional families for individual component equations, and does not require the multivariate normality of the full variable set [26].

The hypothesized causal structure was specified a priori based on the random forest importance rankings (Section 4.4.4) and established agronomic knowledge of forage yield formation in perennial grasses. Genotype (coded as a categorical factor) and year (coded as a categorical factor) were positioned as exogenous upstream variables. Plant height, stem diameter, and acid detergent fiber (ADF) were included as intermediate endogenous variables because these three traits were consistently identified as the top–ranking predictors by the random forest model. Hay yield per plant served as the terminal endogenous response variable. The specified pathways included direct effects from genotype and year to each intermediate trait, direct effects from each intermediate trait to hay yield, direct effects from genotype and year to hay yield (representing unmediated genetic and environmental influences), and the cross–pathway from plant height to stem diameter (reflecting the biological dependency of stem thickening on vertical growth). Each component equation in the piecewise SEM was fitted as a linear model (lm) using ordinary least squares. Model adequacy was evaluated using Fisher’s C statistic, which tests the null hypothesis that there are no missing paths in the specified model [26]. A non–significant Fisher’s C test result (*p* > 0.05) indicates acceptable model fit, meaning that the data do not provide evidence for additional unmodeled pathways. Standardized path coefficients (β) were reported for all pathways to enable a comparison of effect magnitudes across traits measured on different scales. Indirect effects were computed as the product of the constituent standardized path coefficients along each mediation pathway, and total effects were calculated as the sum of direct and indirect effects. The analysis was performed using the piecewiseSEM package (version 2.1.2) in R 4.0.2 [26].

#### 4.4.6. TOPSIS Comprehensive Evaluation

The technique for order preference by similarity to ideal solution (TOPSIS) was employed as a multi–criteria decision–making method to generate a holistic ranking of the five *Bromus inermis* Leyss. accessions by integrating all measured agronomic, yield, and nutritional quality traits into a single composite score [24,25]. The entropy weight method was used to derive objective indicator weights, thereby avoiding the subjectivity inherent in expert–assigned weighting schemes. The computation proceeded through the following four stages, where n denotes the number of germplasm accessions, and m denotes the number of evaluation indicators.

In the first stage (normalization), a proportion matrix *p* was constructed such that each element was calculated as

$$p_{ij}=\frac{x_{ij}}{\sum_{i=1}^{n}\;x_{ij}},i=1,\ldots ,n;j=1,\ldots ,m$$

which represents the original value of accession *i* for indicator *j*. In the second stage (entropy calculation), the information entropy for each indicator was computed as

$$e_{j}=-\frac{1}{\ln n}\sum_{i=1}^{n}\;p_{ij}lnp_{ij}$$

where the convention 0⋅ln0 = 0 was adopted. In the third stage (dispersion coefficient), the degree of divergence for each indicator was obtained as

$$d_{j}=1-e_{j}$$

In the fourth stage (weight determination), the entropy–based weight for each indicator was calculated as

$$w_{j}=\frac{d_{j}}{\sum_{j=1}^{m}\;d_{j}},with\sum_{j=1}^{m}\;w_{j}=1$$

The entropy–derived weights were then applied to the normalized matrix to produce the weighted normalized decision matrix *V*, where each element *v_ij_* = *w_j_*∙*p_ij_*. From this matrix, the ideal solution *V*^+^ was defined as the vector of the maximum weighted values for benefit–type indicators (plant height, stem diameter, tillers per plant, grass layer height, leaf length, leaf width, dry–to–fresh ratio, fresh forage yield per plant, hay yield per plant, crude protein, crude fat, and relative feed value) and minimum weighted values for cost–type indicators (acid detergent fiber, neutral detergent fiber, and crude ash); the anti–ideal solution *V*^−^ was defined conversely. The Euclidean distance from each accession to the ideal solution (*D*i^+^) and to the anti–ideal solution (*D*i^−^) was computed, and the closeness coefficient for each accession was calculated as

$$C_{i}=\frac{D_{i}^{-}}{D_{i}^{+}+D_{i}^{-}},0\leq C_{i}\leq 1$$

where values closer to 1 indicate superior multi–trait performance [24,25]. The TOPSIS computation was implemented using the plyr package (version 1.8.8) in R 4.0.2. All figures were produced using Origin 2021 (OriginLab Corporation, Northampton, MA, USA).

Within the integrated analytical framework, TOPSIS and piecewise SEM served complementary roles corresponding to two distinct analytical stages. TOPSIS served as the first–stage evaluation tool, compressing all measured trait dimensions into a single closeness coefficient to objectively identify the genotype with the best overall multi–trait balance. The piecewise SEM then served as the second–stage mechanistic tool, elucidating through which trait–mediated regulatory links the top–ranked genotype achieved its superior and stable performance across years. This two–stage design ensured that the causal interpretation provided by SEM was anchored to an empirically justified genotype ranking rather than an arbitrary selection.

## 5. Conclusions

Over three consecutive years of field trials, we evaluated four *Bromus inermis* Leyss. accessions and one control cultivar (WUSU) under alpine conditions on the Qinghai–Tibet Plateau. Year effects were statistically significant across all three production years, reflecting the combined influence of developmental stage and annual environmental variation on trait expression; however, the magnitude of the genotype × year interaction differed markedly among years. Trait expression was attenuated by developmental immaturity in year 2, maximized by the convergence of physiological maturity and favorable growing–season precipitation in year 3, and modulated downward by rhizome aging associated with the sod–bound phenomenon in year 4. Consequently, the third growing season represented a critical window in which developmental readiness and environmental favorability converged the most effectively to maximize forage yield and quality, rather than being the only year in which environmental effects were operative. Among all tested accessions, genotype 4–4 emerged as the top–ranked material, consistently outperforming others in both yield and nutritional quality across all production years, and therefore represents a promising candidate for further multi–site validation and forage improvement programs targeting high–altitude environments.

## Figures and Tables

**Table 1:** Independent–sample *t*–tests comparing the two clusters identified by K–means analysis (Group 1: genotypes 3–12 and 4–4; Group 2: genotypes WUSU, 1–10 and 2–10) for major agronomic, yield, and nutritional traits.

Indicators	Plant Height (cm)	Stem Diameter (mm)	Leaf Length (cm)	Leaf Width (cm)	Grass Height (cm)	Tiller Number per Plant	Hay Yield (kg·hm^−2^)	Fresh Forage Yield (kg∙hm^−2^)	Dry–to–Fresh Matter Ratio	Ash (%)	CP (%)	CF (%)	ADF (%)	NDF (%)	RFV
Group 1	108	4.0	15.0	0.8	50.9	186	1627	4445	0.4	7.8	4.7	43.7	37.4	4.0	136
Group 2	97.0	2.8	12.9	0.8	45.1	157	1057	4107	0.3	8.7	4.2	55.8	35.2	3.2	120
*t*	1.4	4.6	1.2	−0.4	2.6	1.7	3.5	0.8	6.8	−3.9	3.6	3.2	−4.9	3.2	5.1
*p*	0.17	0.00	0.30	0.70	0.01	0.10	0.001	0.50	0.00	0.00	0.001	0.003	0.00	0.002	0.00

**Table 2:** Coefficient of variation for major yield and quality traits among genotypes over three years.

Traits	Coefficient of Variation				
	WUSU	1–10	2–10	3–12	4–4
Plant height	0.33	0.30	0.29	0.30	0.25
Stem diameter	0.34	0.26	0.29	0.24	0.22
Leaf length	0.44	0.54	0.51	0.46	0.58
Leaf width	0.25	0.51	0.41	0.42	0.39
Grass height	0.17	0.23	0.21	0.17	0.14
Tiller number per plant	0.40	0.36	0.37	0.31	0.47
Dry–to–fresh matter ratio	0.22	0.21	0.22	0.20	0.11
Hay yield	0.24	0.24	0.61	0.38	0.34
Fresh forage yield	0.39	0.42	0.42	0.43	0.37
Ash	0.10	0.09	0.12	0.08	0.14
CP	0.13	0.16	0.09	0.15	0.11
ADF	0.09	0.06	0.03	0.10	0.30
NDF	0.07	0.05	0.06	0.06	0.06
CF	0.34	0.26	0.27	0.26	0.20
RFV	0.09	0.06	0.06	0.04	0.10

**Table 3:** Weights of all indicators.

Indicators	Plant Height	Stem Diameter	Leaf Length	Leaf Width	Grass Height	Tiller Number per Plant	Dry–to–Fresh Matter Ratio	Hay Yield	Fresh Forage Yield	Ash	CP	ADF	NDF	CF	RFV
Weighting coefficient	0.08	0.06	0.06	0.04	0.05	0.09	0.07	0.04	0.06	0.10	0.04	0.08	0.10	0.05	0.09

**Table 4:** Overview of test materials.

Germplasm Number	Plant Height (cm)	Tiller Number (per Plant)	Fresh Grass Yield (t·hm^−2^)	Growth Adaptation
1–10	60–140	90–220	20–60	Adapted to high altitude, low temperature, and strong radiation.
2–10	60–140	90–200	20–60	Adapted to high altitude, low temperature, and strong radiation.
3–12	70–140	100–200	20–60	Adapted to elevations above 3000 m on the Qinghai–Tibet Plateau.
4–4	80–150	100–300	30–70	Adapted to elevations above 3000 m; maximum yield achieved in production years 3–4.
WUSU	80–150	100–200	20–60	Can be cultivated at altitudes of 600–2500 m in regions with abundant rainfall and warm climates.

## Data Availability

The original contributions presented in this study are included in the article/Supplementary Material. Further inquiries can be directed to the corresponding authors.

## Supplementary Materials

The following supporting information can be downloaded at: https://www.mdpi.com/article/10.3390/plants15101547/s1, Table S1: Analysis of variance (ANOVA) examining the influence of cultivar and planting year on agronomic characteristics in *Bromus inermis*; Table S2: Two-way ANOVA assessing the impacts of cultivar and planting year on the nutritional characteristics of *Bromus inermis*.
