# Supplementary material for: Multi–Year Stability Assessment of Agronomic Performance, Yield and Nutritional Quality of Bromus inermis Genotypes in Qinghai Lake Region
Source: Plants (Basel). 2026 May 19;15(10):1547. doi: 10.3390/plants15101547 (PMC13210550; doi:10.3390/plants15101547)
Supplement: Supplementary file 1 [file plants-15-01547-s001.zip › plants-4264300-supplementary.pdf]

Table S1. Analysis of variance (ANOVA) examining the influence of cultivar and planting year on agronomic characteristics in *Bromus inermis*

**Trait**	**Factor**	**Sum of squares**	**F**	***P***
Stem diameter	Genotype	27.6	83.8	<0.000
	year	21.1	259	<0.000
	Genotype × year	6.2	19.0	<0.000
Plant height	Genotype	4176	28.0	<0.000
	year	8629	232	<0.000
	Genotype × year	802	5.4	0.002
Tiller number per plant	Genotype	34979	28.0	<0.000
	year	68519	219	<0.000
	Genotype × year	21431	17.1	<0.000
Grass height	Genotype	829	26.7	<0.000
	year	1075	139	<0.000
	Genotype × year	220	7.1	<0.000
Leaf length	Genotype	689	28.2	<0.000
	year	903	148	<0.000
	Genotype × year	124	5.1	0.003
Leaf width	Genotype	2.1	16.3	<0.000
	year	0.53	16.7	<0.000
	Genotype × year	1.4	11.0	<0.000

Note: $P > 0.05$ indicated non-significance, $P < 0.05$ indicated significance, and $P < 0.01$ indicated extreme significance. The F-value reflected the overall significance level of the regression equation that was fitted; a higher F-value implied a more substantial difference among the groups.

Table S2. Two-way ANOVA assessing the impacts of cultivar and planting year on the nutritional characteristics of *Bromus inermis*

**Trait**	**Factor**	**Sum of squares**	**F**	***P***
Crude ash (%)	Genotype	20	13	<0.000
	year	0.40	1.0	0.33
	Genotype × year	3.0	1.9	0.12
Crude protein (%)	Genotype	11.7	28.0	<0.000
	year	2.9	27.4	<0.000
	Genotype × year	2.4	5.7	0.002
Acid detergent fiber (%)	Genotype	333	12.0	<0.000
	year	705	102	<0.000
	Genotype × year	130	4.7	0.005
Neutral detergent fiber (%)	Genotype	299	6.60	0.001
	year	28.7	2.53	0.12
	Genotype × year	95.1	2.10	0.11
Crude fat (%)	Genotype	21.36	132	<0.000
	year	5.79	144	<0.000
	Genotype × year	0.78	4.9	0.004
Relative feed value (%)	Genotype	2369	55.6	<0.000
	year	1973	185	<0.000
	Genotype × year	950	22.3	<0.000
